# Latency period and early initiation of breastfeeding in term premature rupture of membrane in Southern Ethiopia, 2017

**DOI:** 10.1186/s13052-019-0662-6

**Published:** 2019-06-07

**Authors:** Yinager Workineh, Teklemariam Gultie

**Affiliations:** 1 Department of child health Nursing, College of Medicine and Health science, Bahir Dar University, Bahir Dar, Ethiopia; 2 Department of Midwifery, College of Medicine and Health science, Arba Minch University, Arba Minch, Ethiopia

**Keywords:** Latency period, Premature rupture of membrane, Early initiation of breastfeeding

## Abstract

**Background:**

World Health Organization recommended timely initiation of breastfeeding within the first hour of delivery. Less than half of newborn babies (43%) receive the benefits of immediate breastfeeding in the world. In East Africa and Ethiopia, the prevalence of early initiation of breastfeeding was 61.82 and 73%, respectively. But, the prevalence of early initiation of breastfeeding was not assessed in relation to the duration of term premature rupture of the membrane in Ethiopia. Therefore, the aim of this study was to assess the effect of the latency period of term premature rupture of the membrane on early initiation of breastfeeding in Southern Ethiopia, 2017.

**Methods:**

The study was conducted in Southern Ethiopia public hospitals by using facility based prospective follow up study from 20th February to 20th August 2017. Then, based on the duration of latency period of term premature rupture of the membrane, 98 and 294 mothers with prolonged and short latency period were followed until the initiation of breastfeeding respectively. Logistic regression analysis was performed to see the association between predictor and outcome variables. Adjusted odds ratio, with 95% CI, was calculated for each independent variable to check the adjusted association between independent variables and dependent variable. The statistical significance was set at *P* < =0.05.

**Results:**

From a total of 91 mothers with prolonged latency period of premature rupture of membrane, 66.0% of them initiated breastfeeding after 1 h of birth. One the other hand, from 289 women with short latency period, 65.7% of them initiated breastfeeding within 1 h of delivery. The odds of initiation of breastfeeding within 1 h of delivery was higher in mothers with a short latency period of term premature rupture of membrane as compared to a prolonged latency period (AOR = 4.169: 95% CI; [1.933, 8.991]). Other variables such as educational status, wealth index, and place of residence were also independent predictors of initiation of breastfeeding.

**Conclusion:**

This study pointed out that women with short latency period of premature rupture of the membrane were more likely to initiate breastfeeding within an hour of delivery than women with prolonged latency period. Therefore, this finding suggested that women with prolonged premature rupture of membrane need special attention to increase early initiation breastfeeding.

## Background

World Health Organization recommended timely initiation of breastfeeding within the first hour of delivery [1]. Provision of a mother’s 1st breast milk, which contains colostrum, protects the newborn from diseases. Early and uninterrupted initiation of breastfeeding encourages bonding between the mother and her newborn. It also increases the production of regular breast milk and the overall duration of breastfeeding [2].

Previous evidence showed that initiation of breastfeeding within 1 h of birth reduces the baby’s risk of death by 44% [3]. On the other hand, infants who initiated breastfeeding after 2–23 h of birth had a 33% risk of mortality as compared to infants who initiated breastfeeding ≤1 h after birth [4]. A similar study conducted in sub-Saharan countries indicated that early initiation of breastfeeding (EIBF) and exclusive breastfeeding practices brought a lower prevalence of diarrhoea among children aged 0–23 months [5].

Worldwide, less than half of newborn babies (43%) receive the benefits of immediate breastfeeding [3]. The prevalence of EIBF in West Africa, East Africa, Central Africa, and Southern Africa was 46.94, 61.82%, 37.84 and 69.31% respectively. Guinea (16.54%) in West Africa, Kenya (30.38%) in East Africa, Congo Brazzaville (24.10%) in Central Africa, and Lesotho (66.24%) in Southern Africa had lowest prevalence of EIBF [6]. The prevalence of EIBF in the least developed countries was 53% [3]. In Ethiopia, the 2016 Ethiopian demographic survey [7] showed that a 73% of newborns were breastfed within 1 h of birth. From all infants, 92% of them were breastfed within 1 day of birth [7].

Several studies had shown that being unmarried, delivering via cesarean section, lack of counseling on EIBF, having lower educational level, preferring of male sex and facing obstetric problems were the main determinants of delayed initiation breastfeeding [8, 9]. Caesarean section delivery, home delivery, and being primiparous were also inversely associated with EIBF [10, 11].

On the other hand, age at birth, educational status, urban residency, frequent antenatal visits, and singleton birth were the main factors that increase EIBF [10]. Another study reported that EIBF was affected by husband support and the role of health workers [12]. The study conducted by Liben and Yesuf also stated that women resided in urban areas and attended the formal education were positively associated with EIBF [11].

Even though, 8 to 10% term pregnancies experienced spontaneous PROM [13], there was no study done before that investigates the relation between duration of PROM and EIBF. This PROM was also associated with abnormal newborn outcome as a result of asphyxia, meconium aspiration and suffocation [14].

In spite of the above challenge of PROM, the relation between the length of the latency period of PROM and EIBF was not given special attention before. Dealing with the relationship of duration of PROM and EIBF is very vital to give direction for designing of strategies for timely initiation of breast feeding among mothers with PROM in Ethiopian context. Also, it will narrow the evidence gap, and provide fruitful findings for responsible bodies to avoid mitigating factors of EIBF in the case of PROM. Therefore, the aim of this study was to assess the effect of the latency period of term premature rupture of membrane on EIBF in Southern Ethiopia, 2017.

## Methods

### Study setting, design and period

The study was conducted in Southern Ethiopia specifically in Gamo Gofa, Segen people’s and South Omo public hospitals. The administrative center of Gamo Gofa, Segen people’s and South Omo zone is Arba Minch, Gumaidie and Jinka respectively. Arba Minch, Chencha, Sawula, Gidolie and Jinka hospitals are the general and referral hospitals which provides services for the mothers and neonates in these areas. Facility based prospective follow up study was conducted in these hospitals from 20th February to 20th August 2017.

### Participant selection and exclusion

All mothers who were diagnosed as term PROM by physicians were recruited as a study subject in the labor admission room. Then, selected admitted term PROM mothers were observed based on the treatment decision of physicians from time of admission to initiation of labour. After determination of the duration of PROM, the mothers were also observed up to the initiation of breastfeeding.

However, women who did not remember their exact time of initiation of membrane rupture, diagnosed for preterm PROM in the current pregnancy, and who reached term at the entry time were excluded. In addition, women with gestational age of < 37 and > 42 weeks were excluded from the study. Women experienced emergency delivery due to medical and obstetrics complication, and mothers with sick baby were excluded from the study.

### Sample size determination and sampling technique

The sample size was calculated using Epi-Info version 3.5.1 statistical software package by considering the following assumptions. The assumptions used were 95% confident interval (CI), 85% power of the study, 2.2 odds ratio, 50% of unexposed outcome (since no study done before), 1:3 case to control ratio and 10% non-response rate. The final sample size was 98 for exposed and 294 for un-exposed groups with a total sample of 392.

The five public hospitals namely Arba Minch, Chencha, Sawula, Gidole and Jinka were used as a study site. These Hospitals are a flagship zonal and district hospitals that serve the community living in respective towns and nearby districts. Then, based on the number of clients who visited each hospital during the previous 1 year, the total sample size was proportionally allocated to each hospital. Finally, woman diagnosed as term PROM during a study period, were followed until initiation of breastfeeding.

### Measurements

Five trained midwives collected data from the participants using interviewer administered questionnaire, observational checklist and physical measurements. The questionnaire consists of socio-demographic variables, obstetric history, birth preparedness, complication readiness and initiation of breastfeeding.

The observational checklist was used to gather data on fetal 1st and 5th minute Apgar score, which is classified as with asphyxia (< 7 scores) and no asphyxia (> = 7 scores). This value is the commutative score of skin color (coded as 0 = blue-pale, 1-body pink-limb pale and 2 = body and limb pink), muscle tone (coded as 0 = absent-limp, 1 = some flexion of extremities and 2 = active), heartbeat (coded as 0 = absent, 1 = < 100 and 2= > 100), respiration (coded as 0 = absent, 1 = week cry and 2 = strong cry) and response to stimuli (coded as 0 = absent, 1 = slight and 2 = good sneeze).

Inter pregnancy interval, length of labor, PROM and the latency period of PROM was measured by time. Interpregnancy interval (IPI) is the interval between the most recent previous childbirth and conception of the index pregnancy as reported by the mother. It was classified as optimal (2–5 years) and short (below 2 years) IPI. Length of labor is the time from initiation of labor to the end of the third stage of labor. It was categorized as > = 24 h and < 24 h. Rupture of membrane before initiation of labor was classified as preterm (occurred before 37 weeks of gestation) and term PROM (occurred after 37 weeks of gestation) based on gestational age. The latency period of PROM, main predictor variable, was measured from time of reported rupture of membranes until time of initiation of labor in hours. It was categorized as prolonged (> = 24 h) and short (< 24 h) latency period.

Physical measurements were used to obtain data on mid upper arm circumference (MUAC) of mothers, gestational age of mothers and weight of newborn. In this regard, MUAC of each woman was measured at the midpoint between the tips of the shoulder and elbow of the left arm using non-elastic, non-stretchable MUAC tapes. Measurements were recorded to the nearest 0.1 cm. In this study, poor nutritional status of the mother, defined as MUAC < 23 cm [15]. Weight of newborn was measured by been balance immediately after delivery and classified as low (< 2500 g) and normal (> = 2500 g) birth weight.

Initiation of breastfeeding, the outcome variable, was classified as early (initiation of breastfeeding within 1 h) and delayed (initiation of breastfeeding after 1 h of delivery) during the follow up period.

Birth preparedness and complication readiness (BPCR) was measured by using the mean value of plan to place of delivery, plan to skilled assistant, saved money for obstetric emergency, plan to mode of transport, plan to blood donor, and detect early signs of an emergence. Based on these variables, BPCR was classified as prepared (score > = the mean) and non-prepared (score < the mean).

### Data quality assurance

To make sure consistency, questionnaire was prepared in English and translated to Amharic version, and re translated back to English. Pre-test of the tool was performed outside the study area and adjustment was done based on the result. Intensive training for data collectors was given for 2 days. To insure quality, the data collection process was supervised continuously. Finally, the collected data were carefully checked on daily basis for completeness, outliers and missing values as well as consistencies.

### Data analysis

The collected data were entered, cleaned, coded and analysis using SPSS version 20. Frequency distribution for selected variables was performed. Cleaning of the data was performed before analysis. To check the statistical significance between the dependent and independent variables, chi-square tests was performed. In order to know the crude association between latency period of PROM and EIBF, crude odds ratio (COR) of EIBF with 95% Confidence interval (CI) were calculated. Those variables, with *P* < 0.2 from the bivariate analysis were considered for multivariable logistic regression.

Logistic regression analysis was performed to see the association between predictor and outcome variables. Adjusted odds ratio (AOR) with 95% CI was calculated for each independent variable to check the adjusted association between independent variables and EIBF. Finally, the statistical significant was set at *P* < 0.05.

## Results

During the 6 month period, 392 participants with term PROM were recruited as a study participant from five hospitals. However, 12 of them were excluded in the follow up time due to referral reason. Finally, we observed 91 and 289 women with short and prolonged latency period of term PROM respectively.

Participants exposed to prolonged latency of PROM tended to be illiterate (*p* = 0.00), non-governmental employee (*P* = 0.00), have very poor wealth index (*P* = 0.00) and resigned in rural area (*p* = 0.00) as compared to women with short latency period (Table 1).

**Table 1:** Distribution of selected variables among women with prolonged PROM and short PROM in selected hospitals, Southern Ethiopia, 2017

Variables	Short latency group (*n* = 289) *n*	Prolonged group(*n* = 91) *n*	*p-value
Maternal age			
15–19 year	36	8	0.799
20–24 year	122	28	0.836
25–29 year	82	26	0.752
30–34 year	27	12	0.746
35–39 years	20	17	0.970
40 + years	2	0	1
Occupation			
Housewife	194	57	1
Farmer	27	6	**0.024**
Government Employee	20	14	**0.000**
Merchant	39	9	0.351
Non-government employee	9	5	**0.000**
Educational status			
Non-educated	59	15	**0.000**
Read and write	24	7	**0.132**
Grade 1–6	93	34	**0.003**
Grdae 7–8	50	8	**0.015**
Grade 9–12	36	10	0.481
Above grade 12	27	17	1
Marital status			
Married	280	90	0.35
Unmarried	9	1	
Residence			
Urban	106	21	**0.000**
Rural	183	70	
Wealth index			
Very poor	37	16	
Poor	56	17	**0.000**
Rich	58	18	**0.000**
Medium	63	21	**0.000**
Very rich	75	19	**0.000**
IPI			
Short	106	21	**0.000**
Optimal	183	70	
Utilization of ANC			
No	28	4	**0.025**
Yes	261	87	
Level of ANC visit			
One visit	1	1	0.48
Two visit	11	7	0.37
Three visit	38	10	0.78
Four and more	211	69	0.20
Time of initiation of ANC			
Within 4 months	185	54	0.24
After 4 months	77	33	
Husband support			
No	9	1	
Yes	280	90	0.86
BPCR			
Not prepared	280	77	**0.002**
Prepared	9	14	
Gestation			
Singleton	284	84	0.562
Twins	5	7	
Initiation of labour			
Spontaneous	261	47	0.197
Induction	28	44	

*Chi-square test was used to obtain the *p*-value

Bold data are those which are significant

Similarly, non-utilization of the antenatal care (ANC) (*P* = 0.025), short IPI (*p* = 0.00) and not prepared for birth (*P* = 0.002) were higher in exposed women. The two groups did not significantly differ in terms of maternal age, marital status, husband support, level of ANC visit, time of ANC initiation, initiation of labor and multiple gestations (Table 1).

From 91 observed mothers with prolonged PROM in the hospitals, 66.0% of them initiated breastfeeding after 1 h of delivery. Similarly, from all term PROM case women with short latency period, 65.7% of them initiated breastfeeding within 1 h of delivery. This implied that women with short latency period of PROM were more likely to initiate breastfeeding within 1 h of delivery (AOR = 3.715: 95% CI; [2.260, 6.106]) (Table 2).

**Table 2:** Prevalence of early initiation of breastfeeding with short and prolonged duration of latency period of PROM in public hospitals, Southern Ethiopia, 2017

Outcome variable	Groups				*P*-value	COR(95%CI)
	Short latency (*n* = 289)		Long latency (*n* = 91)			
	No	%	No	%		
Initiation of breastfeeding						
Early (within 1 h of delivery)	190	65.7	31	34.0	0.000*	**3.715 (2.260,6.106)**
Delayed (after 1 h of delivery)	99	34.3	60	66.0		

Chi-square test was used to obtain the *p*-value

*Statistically significant at *p* = 0.000

### Risk factors of initiation of breastfeeding

After adjustment of the potential confounders such as educational status, place of residence, wealth index, ANC status, initiation of labor, length of labor, newborn weight and BPCR, the association between latency period and initiation of breastfeeding was remained significant. The odds of initiation of breastfeeding within 1 h of delivery was higher among women with short latency period as compared to mothers with prolonged latency period (AOR = 4. 169: 95% CI; [1.933, 8.991]) (Table 3).

**Table 3:** Duration of latency period of PROM and odds of early initiation of breastfeeding labor in relation to other confounding variables in selected public hospitals, Southern Ethiopia, 2017

Variables	Early initiation of breastfeeding	
	COR(95%CI)	AOR(95%CI)
Educational status		
Non-educated	**0.121 (0.051, 0.283)**	**0.265 (0.100, 0.700)**
Read and write	0.485 (0.189, 1.242)	0.889 (0.373, 2.119)
Grade 1–6	0.336 (0.164, 0.689)	1.620 (0.483, 5.440)
Grdae 7–8	0.365 (0.162, 0.824)	0.517 (0.205, 1.300))
Grade 9–12	0.735 (0.312, 1.730)	1.142 (0.426, 3.058
Above grade 12	1	1
Residence		
Urban	1	1
Rural	**0.084 (0.045, 0.156)**	**0.112 (0.055, 0.225)**
Latency period		
Short	**3.715 (2.260, 6.106)**	**4.169 (1.933, 8.991)**
Prolonged	1	1
Utilization of ANC		
Yes	0.517 (0.232, 1.149)	0.879 (0.319, 2.423)
No	1	1
Wealth index		
Very poor	1	**1**
Poor	**9.128 (4.214, 19.771)**	1.978 (0.859, 4.556)
Rich	**3.636 (1.839, 7.186)**	1.729 (0.746, 4.005)
Medium	**3.370 (1.714, 6.624)**	1.422 (0.637, 3.175)
Very rich	**2.554 (1.311, 4.977)**	**3.635 (1.401, 9.434)**
Initiation of labour		
Spontaneous	**0.712 (0.426, 0.993)**	1.238 (0.535, 2.865)
Induction	1	1
Weigth of child		
> =2500 g	**0.152 (0.056, 0.413)**	0.419 (0.101, 1.743)
< 2500 g	**1**	1
Length of labor		
< 24 h	**0.689 (0.411, 0.957)**	1.300 (0.553, 3.057)
> =24 h	1	1
BPCR		
Not prepared	**0.440 (0.185, 0.993)**	1.187 (0.270, 5.219)
Prepared	1	1

Single model was used to produce the AORs

Adjusted for nine variables shown in the table

Bold data are those which are significant

Educational level, wealth index and place of residence were also an independent predictor of initiation of breastfeeding in multiple logistic regression analysis. The effect of a short latency period on EIBF was increased in the very rich wealth index (AOR = 3. 635: 95% CI; [1.401, 9.434]). But, the effect of latency period on EIBF was decreased in non-educated (AOR = 0. 265: 95% CI; [0.100, 0.700]) and rural residence mothers (AOR = 0. 112: 95% CI; [0.055, 0.225]) (Table 3).

## Discussion

Determination of the effect of the latency period of term PROM on EIBF, by using prospective follow up study design in Southern Ethiopia public hospitals, was the main objective of this study.

On the base of the objective, the current finding indicated that short latency period of PROM was the main predictor of EIBF independent of educational level, place of residence and wealth index. The independent positive effect of short latency period of PROM on EIBF might be occurred as a result of minimal postpartum mood disturbance as compared with prolonged latency period of term PROM. This minimal undisturbed mood in short latency period of PROM would have high maternal-newborn bonding and early skin-to-skin contact as compared with prolonged latency period. Hence, uninterrupted early skin-to-skin contact enhances breastfeeding success during the early postpartum period [16]. Skin to skin contact also induces oxytocin, which antagonizes the flight-fight effect, decreasing maternal anxiety and increasing calmness and social responsiveness [17]. During the early hours after birth, oxytocin may also enhance parenting behaviors [18, 19]. Therefore, EIBF could be higher in short latency period of term PROM as compared with prolonged latency period.

In this study, lower level educational status was inversely associated with EIBF which is similar with the other studies [8, 9]. On the other corner, the study by Yahya and Adebayo, and Liben and Yesuf stated that mother’s educational status was positively associated with EIBF. This could be explained by the fact that low level of education brings limitation of the utilization ANC services [20]. This also leads to low level of women confidence and capability to make decisions about different maternal service utilization [21, 22]. Thus, this might facilitate delayed initiation breastfeeding among these participants.

The residence was the other independent predictor of EIBF in PROM case mothers. Mothers from rural areas were inversely associated with EIBF as compared with mothers from urban area. This finding is similar to the other previous studies EIBF [10, 11, 23]. The reason for this could be due to low level of ANC services utilization, which is an independent preventive factor of EIBF, as evidenced by other previous studies [24, 25]. The low level of ANC service utilization might lead to lack of counseling about EIBF. Intern, lack of counseling about EIBF could bring late initiation of breastfeeding in rural area as compared with urban area [8, 9].

The present study also showed that very rich wealth index was positively associated with EIBF. Similar association had been noted in the past studies [23, 26, 27]. Women from the richest households were more likely to utilize the desirable maternal health services package [28]. Thus, utilization of maternal health service might alert the women to initiation breastfeeding within 1h of delivery.

This research might be subjected to recall bias since participants might not remember and report past events. The other limitation of this study might be selection biases if there are participants who do not know initiation of PROM. The last but not the least limitation of this study was loss to follow up due to referral.

## Conclusion

This study pointed that women with short duration of PROM during follow up time were more likely to initiated breastfeeding within hour of delivery. Therefore, these findings suggested that prolonged PROM case mothers need special attention to increase EIBF.

## Data Availability

Data of this study will be obtained by contacting the corresponding author via this email: workieyenie@gmail.com.com or Tel no. + 251972598612.

## Abbreviations

ANC: Ante Natal Care
AOR: Adjusted Odds Ratio
BPCR: Birth Preparedness and Complication Readiness
CI: Confidence Interval
COR: Crude Odds Ratio
EIBF: Early initiation of breastfeeding
IPI: Interpregnancy interval
MUAC: Mid Upper Arm Circumference
PROM: Premature rupture of membrane
